# Driver mutations in myeloid and lymphoid cells point to multipotent progenitor origin of diverse histiocytic neoplasms^∗^^†^

**DOI:** 10.1016/j.bneo.2025.100074

**Published:** 2025-01-27

**Authors:** Astrid G. S. van Halteren, Paul G. Kemps, Jelske Forma-Borst, Yanling Xiao, Maud A. J. I. van den Oetelaar, Nina U. Gelineau, Robert M. Verdijk, Lydia E. Vos, Koen D. Quint, Cor van den Bos, Eli L. Diamond, Jan A. M. van Laar

**Affiliations:** 1 Clinical Immunology and Allergology Section, Department of Internal Medicine, Erasmus MC University Medical Center Rotterdam, Rotterdam, The Netherlands; 2 Princess Máxima Center for Pediatric Oncology, Utrecht, The Netherlands; 3 Department of Pathology, Leiden University Medical Center, Leiden, The Netherlands; 4 Department of Pediatrics, Leiden University Medical Center, Leiden, The Netherlands; 5 Department of Immunology, Leiden University Medical Center, Leiden, The Netherlands; 6 Department of Immunology, Laboratory Medical Immunology, Erasmus MC University Medical Center Rotterdam, Rotterdam, The Netherlands; 7 Department of Experimental Immunohematology, Sanquin Research, Amsterdam, The Netherlands; 8 Department of Pathology, Erasmus MC University Medical Center Rotterdam, Rotterdam, The Netherlands; 9 Department of Dermatology, Haaglanden Medical Center, The Hague, The Netherlands; 10 Department of Dermatology, Leiden University Medical Center, Leiden, The Netherlands; 11 Department of Neurology, Memorial Sloan Kettering Cancer Center, New York, NY; 12 Department of Immunology, Erasmus MC University Medical Center Rotterdam, Rotterdam, The Netherlands

## Abstract

Histiocytic neoplasms are rare myeloid diseases characterized by MAPK pathway–activating genetic alterations. We investigated their hematopoietic origin, with a focus on non-Langerhans cell histiocytoses. Using droplet digital polymerase chain reaction assays specific for *BRAF*, *MAP2K1*, or *KRAS* alterations detected in histiocytosis lesions, we could trace the same driver mutation to circulating blood cells in 13 of 14 patients. In 9 of 13 patients, the mutations were detected in circulating lymphoid cells, indicating that multipotent progenitors probably acquired these alterations. The 9 patients included 5 adults with single-system disease, including 3 with recurrent cutaneous xanthogranulomas. The presence of long-lived mutated progenitor cells in these 3 patients was supported by the detection of the same *KRAS* or *BRAF* mutation in xanthogranulomas that developed up to 25 years apart. As proof of concept, we traced the driver mutation to circulating CD34^+^ progenitors in 1 of the 3 patients. Distinct secondary mutations in either *KRAS*, *BRAF*, or *ARAF* were identified in separate xanthogranulomas from the same patient, indicating a 2-hit mutational process underlying the formation of these recurrent lesions. Finally, histiocytes and B cells harboring the same *KRAS* mutation were identified in the unifocal Langerhans cell sarcoma lesion of the only patient without circulating mutated cells. Together, these data point toward multipotent hematopoietic progenitors as the cell of origin of both single-system and multisystem histiocytoses.

## Key Points

• Driver mutations were traced to myeloid and lymphoid lineage cells in patients with single-system histiocytosis.
• Recurrent xanthogranulomas may arise through second hits acquired by the progeny of long-lived *BRAF*- or *KRAS*-mutated multipotent progenitors.

## Visual Abstract

**Figure:**
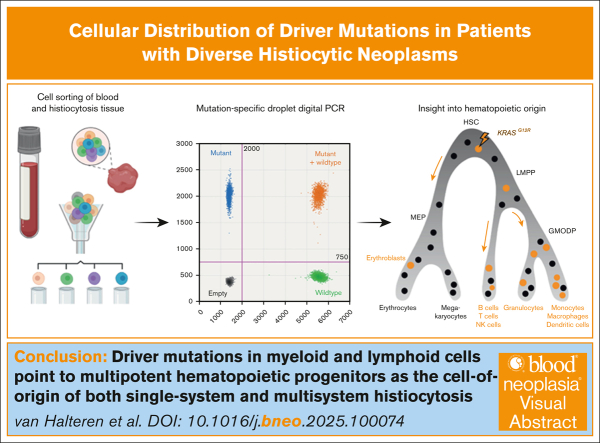

## Introduction

Histiocytic neoplasms are rare diseases frequently characterized by somatic mutations in genes of the MAPK signaling pathway.^1^, ^2^, ^3^, ^4^, ^5^, ^6^, ^7^ They can be categorized into different subgroups,^8^^,^^9^ including the Langerhans (L) group, cutaneous and mucocutaneous (C) group, Rosai-Dorfman (R) group, and malignant (M) group.^8^ Langerhans cell histiocytosis (LCH) and Erdheim-Chester disease (ECD) are both categorized in the L group.^8^ In patients with systemic LCH and/or ECD, the *BRAF*^*V600E*^ mutation can be identified in CD34^+^ hematopoietic stem/progenitor cells (HSPCs) and their offspring.^10^, ^11^, ^12^, ^13^, ^14^ Moreover, mutated bone marrow cells of patients with LCH or ECD formed histiocytic lesions in xenograft mouse models.^12^^,^^15^^,^^16^ These findings provided evidence that mutated HSPCs from patients with LCH and/or ECD can drive their disease.

In the context of LCH, it has been postulated that the extent and severity of the disease are determined by the stage of differentiation of the hematopoietic precursor cell in which the driver mutation arises.^10^^,^^17^ In this model, mutations in HSPCs give rise to high-risk multisystem LCH (characterized by liver, spleen, and/or bone marrow involvement), whereas the same mutations in circulating or tissue-restricted myeloid precursors give rise to low-risk LCH.^10^ By identifying *BRAF*-mutated myeloid and/or lymphoid cells in the blood of several patients with single-system LCH, we already demonstrated that this model is an oversimplification.^13^ In this study, we expand our analysis to other histiocytoses, demonstrating driver mutations in myeloid and lymphoid cells in most patients and across histiocytosis subtypes.

## Methods

### Patients and samples

Peripheral blood and/or fresh lesional tissue-derived mononuclear cells were obtained from 13 adults with diverse histiocytic neoplasms and 1 child with Langerhans cell sarcoma (LCS). Methods for cell isolation, cryopreservation, and sorting have been reported previously.^13^^,^^17^ Exemplary gating strategies are provided in supplemental Figure 1; validation of sorted cell purity is provided in supplemental Figure 2. Clinical data are provided in supplemental Table 1. This study was approved by the institutional review board of Erasmus MC University Medical Center Rotterdam (MEC-2020-0352) in accordance with the Declaration of Helsinki; all patients consented for biobanking of leftover samples, which we obtained after approval by biobank committees of involved institutions.

### Mutation detection

DNA was extracted from total peripheral blood mononuclear cells, granulocytes, or sorted cells using Qiagen QIAamp Micro kits and analyzed using custom-designed or catalog-listed mutation-specific droplet digital polymerase chain reaction assays, as described previously.^13^ The specificity and reproducibility of these assays are demonstrated in supplemental Figures 3 and 4.

## Results

**Figure 1.**
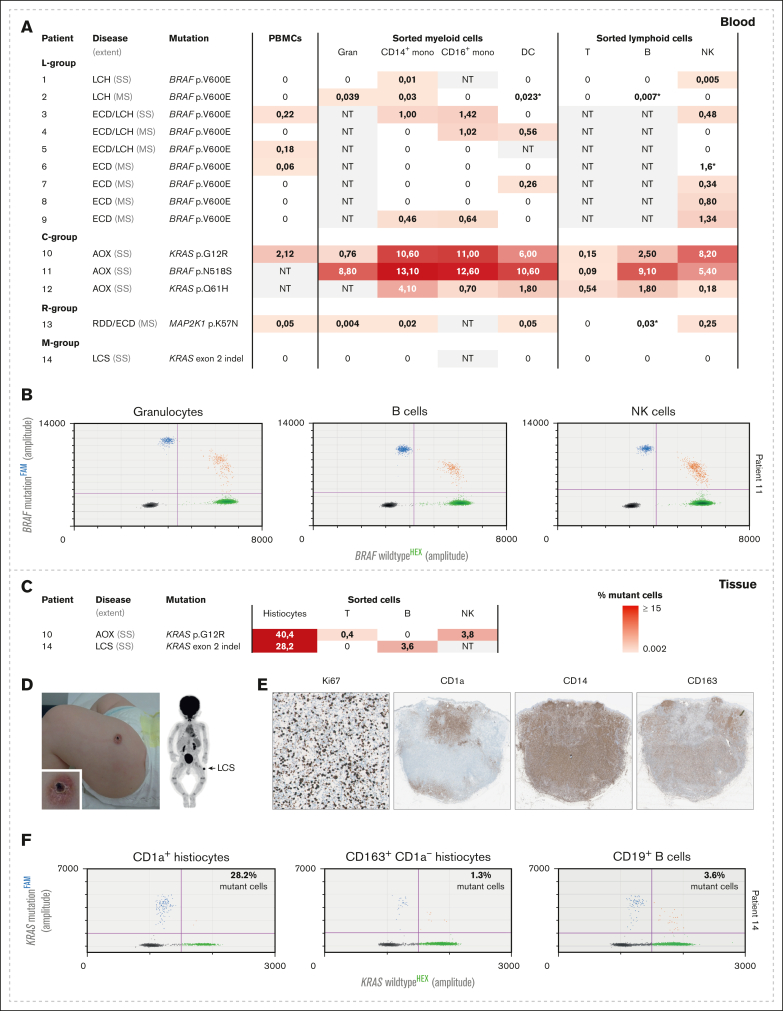
**Molecular analysis of blood- and tissue-derived hematopoietic cell populations.** (A) Frequency of mutation-carrying cells among total PBMCs or lineage-committed cells isolated from blood samples of patients 1 to 14. Every horizontal row represents 1 patient; the shade of red indicates the percentage of mutant cells (with 0% in white and ≥15% in dark red). Data were generated with QuantaSoft software (Bio-Rad). Percentages were calculated by multiplying the fractional abundance by 2, as these are all heterozygous mutations. Gating strategies for lineage cell sorting are found in supplemental Figure 1A. ∗Less than 3 droplets with mutation-specific amplicons; therefore, these samples are considered not unequivocally positive. (B) Exemplary droplet digital polymerase chain reaction (ddPCR) plots revealing the detection of the *BRAF*^*N518S*^ mutation in gran, B, and NK cells isolated from the peripheral blood of patient 11. Droplets containing only mutation-specific PCR products are depicted in blue, whereas droplets containing only wild-type *BRAF* amplicons are depicted in green. Orange droplets contain both PCR products; gray droplets contain no PCR products. Assay validation results and further details can be found in supplemental Figure 3. (C) Frequency of mutant cells among sorted subsets from lesional tissue from patients 10 and 14, as indicated. (D) Photographs (left) and positron emission tomography image (right) showing the isolated tumor in the left upper thigh of patient 14, who was diagnosed with LCS. (E) Photomicrographs of immunostained tissue slides of the tumor in patient 14, which demonstrated a high Ki67 proliferation index and a complex immunophenotype. The tumor was characterized by a central core containing CD1a^+^ cells, surrounded by many CD1a^−^ CD163^+^ histiocytes; CD14 stained cells from both populations. (F) ddPCR plots depicting the detection of the *KRAS* exon 2 indel in CD1a^+^ histiocytes, CD1a^−^ histiocytes, and CD19^+^ B cells sorted from lesional tissue in patient 14. The exact gating strategy used for cell sorting is depicted in supplemental Figure 1B. AOX, adult-onset xanthogranuloma; B, B cells; DC, dendritic cells; gran, granulocytes; indel, insertion-deletion; mono, monocytes; MS, multisystem; NK, natural killer; NT, not tested; PBMC, peripheral blood mononuclear cell; RDD, Rosai-Dorfman disease; SS, single system; T, T cells.

We analyzed the cellular distribution pattern of driver mutations in 14 patients (Figure 1). Blood and tissue samples were obtained from patients with active histiocytic disease; only 3 of 14 had received prior systemic therapy (supplemental Table 1). The full spectrum of histiocytic disorders was captured, including histiocytoses from the L, C, R, and M groups,^8^ and investigated mutations included *BRAF*^*V600E*^, *BRAF*^*N518S*^, *MAP2K1*^*K57N*^, and 3 *KRAS* alterations. Furthermore, 8 patients had multisystemic disease, whereas 6 had single-system histiocytosis. These 6 patients included 1 adult with unifocal bone LCH (patient 1), 1 adult with multifocal bone ECD/LCH (patient 3), 3 adults with recurrent cutaneous xanthogranulomas (patients 10-12), and 1 child with unifocal cutaneous LCS (patient 14).

Driver mutations were detected in unfractionated peripheral blood mononuclear cells of 5 of 12 tested individuals and in peripheral blood subsets of 11 of 14 patients (Figure 1A-B). In 9 of 11 patients with mutated lineage cells, the mutations could be traced to lymphoid cells, with highest frequency in B cells and natural killer cells. Among the myeloid cells, the frequency of mutated cells was often highest in monocytes. There were 4 patients who had mutated granulocytes. In patient 10, *KRAS*^*G12R*^ was detected in both CD141^+^ and CD1c^+^ classical dendritic cell subsets (supplemental Figure 3) and detected at very similar variant allele frequencies in matched lineage cells isolated at 2 different time points, with an interval of more than a year (supplemental Figure 5).^11^^,^^17^^,^^18^

**Figure 2.**
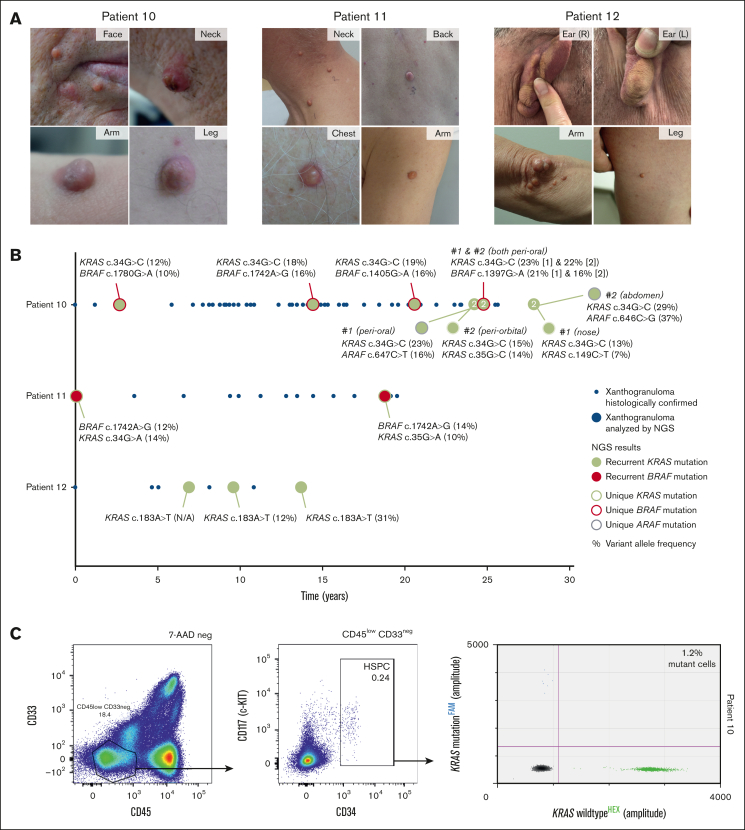
**Analysis of patients with recurrent cutaneous xanthogranulomas.** (A) Photographs depicting the cutaneous xanthogranulomas in patients 10 to 12, which developed during multiple decades at varying anatomic locations. (B) Graphic representation showing the development of multiple cutaneous xanthogranulomas over time in these patients. Note that only xanthogranulomas confirmed by histology are depicted, whereas many additionally resected lesions were not sent for pathologic evaluation and are therefore not depicted. Xanthogranulomas that were analyzed by next-generation sequencing (NGS) are depicted by large (green or red) colored dots; the color of the dots and their outline indicate the detected somatic mutations. These mutations are also specified. Percentages indicate the variant allele frequencies of detected mutations. In patient 10, NGS was sometimes performed on 2 separate xanthogranulomas resected at the same time point; these instances are indicated by a “2” in the large dot. (C) Gating strategy used to isolate CD34^+^ HSPCs from live CD45^bright/dim^ PBMCs in patient 10 (left and middle panels). DNA extracted from these cells was subsequently analyzed using *KRAS* p.G12R-specific ddPCR, demonstrating the presence of the driver mutation in 1.2% of flow-sorted progenitors (right panel). Neg, negative.

Surprisingly, the frequency of mutant cells was substantially higher in the 3 patients with recurrent cutaneous xanthogranulomas (patients 10-12) than in the patients with LCH, ECD, and/or Rosai-Dorfman disease. These 3 adults had developed multiple xanthogranulomas during several decades and in diverse anatomic locations (Figure 2). All 3 patients had normal complete blood counts and leukocyte differentials, without monocytosis. Because they did not have extracutaneous lesions, as supported by positron emission tomography in all 3 patients, none had ever received systemic treatment. Instead, symptomatic lesions had been resected from time to time. To further investigate the molecular pathogenesis of these lesions, next-generation sequencing was performed of up to 9 separate xanthogranulomas from the same patient. In all 3 patients, the exact same *KRAS* or *BRAF* mutation was detected in each skin lesion analyzed, with intervals measuring up to 25 years for patient 10, 19 years for patient 11, and 6.5 years for patient 12. In addition, distinct secondary mutations were identified in separate xanthogranulomas of patients 10 and 11, including unique *KRAS*, *BRAF*, and *ARAF* mutations (Figure 2B). In patient 10, the *KRAS* p.G12R mutation could be traced to CD34^+^ HSPCs (Figure 2C) and glycophorin A^+^ CD71^+^ CD117^−^ erythroblasts (supplemental Figure 6) isolated from the blood.

Besides subsets from the peripheral blood, we evaluated sorted subsets from lesional tissue obtained from patients 10 and 14 (Figure 1C). This analysis revealed *KRAS* mutations in myeloid and lymphoid cells in both patients, including the child with LCS in whom we did not identify *KRAS*-mutated cells in the blood (Figure 1A; supplemental Figure 3). This child had a single tumor in the left upper leg with a high Ki67 proliferation index and a complex immunophenotype, which was classified as LCS (Figure 1D-E). In addition to multiple copy number alterations, the tumor harbored a novel insertion-deletion in *KRAS* exon 2. Using a custom-designed droplet digital polymerase chain reaction assay, we detected this mutation in CD1a^+^ histiocytes, CD1a^−^ histiocytes, and CD19^+^ B cells sorted from the LCS lesion (Figure 1F; supplemental Figure 1).

## Discussion

Our study reveals identical driver mutations in myeloid and lymphoid cells in patients with diverse histiocytic neoplasms, pointing toward multipotent progenitors as the cell of origin of these neoplasms. Importantly, this finding was not restricted to patients with multisystem disease but also applied to patients with single-system histiocytosis of the L, C, or M groups. Thus, the clinical extent of histiocytic neoplasms cannot simply be explained by the stage of differentiation of the hematopoietic cell of origin.

Although cell sorting may not always achieve 100% purity, our results were consistent across patients and different experiments (supplemental Figures 4 and 5). We identified mutation-carrying natural killer cells in 9 adult patients, substantiating previous findings in several children with LCH.^13^^,^^19^ In addition, we identified mutated granulocytes in 4 patients, extending earlier findings in few other cases.^12^^,^^19^ Most strikingly, *KRAS* or *BRAF* mutations were detected in all sorted subsets from 3 patients with recurrent cutaneous xanthogranulomas, suggesting the involvement of an uncommitted HSPC. This mutated HSPC was active during multiple decades (Figure 2). The recurrent xanthogranulomas probably arose through the acquisition of distinct secondary mutations in the progeny of this mutated progenitor. In patient 10, xanthogranulomas resected at the same time point from nearby facial locations even had distinct secondary mutations, suggesting that the mutations were acquired locally. Potentially, the secondary mutations were induced by UV radiation, as they often comprised UV-associated C>T/G>A mutations in a dipyrimidine context.^20^ Accordingly, the xanthogranulomas frequently manifested in sun-exposed skin (eg, the face). To our knowledge, such a 2-hit mutational process underlying recurrent histiocytic lesions has not been demonstrated previously. In patient 12, we likely failed to detect the secondary mutations because the involved genes were not covered by our next-generation sequencing panel or because the mutations were fusions.^5^

In addition to the bone marrow, hematopoietic progenitor cells may be present in the blood or tissue.^21^^,^^22^ Previously, we demonstrated that pediatric patients with LCH have an increased frequency of circulating CD34^+^ myeloid-committed progenitors compared with healthy controls.^13^ We now provide the first evidence of circulating CD34^+^ progenitors harboring a histiocytosis-associated driver alteration in a patient with isolated cutaneous histiocytosis (Figure 2C). In addition, we hypothesize that a multipotent progenitor recruited to the tissue might have been the cell of origin in patient 14, in whom we did not identify any *KRAS*-mutated cells in the blood but detected *KRAS*-mutated histiocytes and B cells in the LCS lesion. Notably, the tumor developed at the site of a previous vaccination, which might have provided the required inflammatory environment for the recruitment and/or local expansion of the neoplastic progenitor.

In conclusion, we traced oncogenic driver mutations to myeloid and lymphoid cells in patients with diverse histiocytic neoplasms, including those confined to a single organ. Mutated progenitors may be long lived, warranting careful follow-up of patients with histiocytosis. Future studies need to identify factors beyond the stage of differentiation of the hematopoietic cell of origin that determine the extent and severity of histiocytic neoplasms.

Conflict-of-interest disclosure: E.L.D. discloses unpaid editorial support from Pfizer Inc and serves on an advisory board for Opna Bio, both outside the submitted work. The remaining authors declare no competing financial interests.
