# Supplementary material for: Driver mutations in myeloid and lymphoid cells point to multipotent progenitor origin of diverse histiocytic neoplasms
Source: Blood Neoplasia. 2025 Jan 27;2(2):100074. doi: 10.1016/j.bneo.2025.100074 (PMC12067900; doi:10.1016/j.bneo.2025.100074)
Supplement: Supplemental Figures and Table [file BNEO_NEO-2024-000430-mmc1.pdf]

Supplemental Figure 1. Exemplary gating strategies

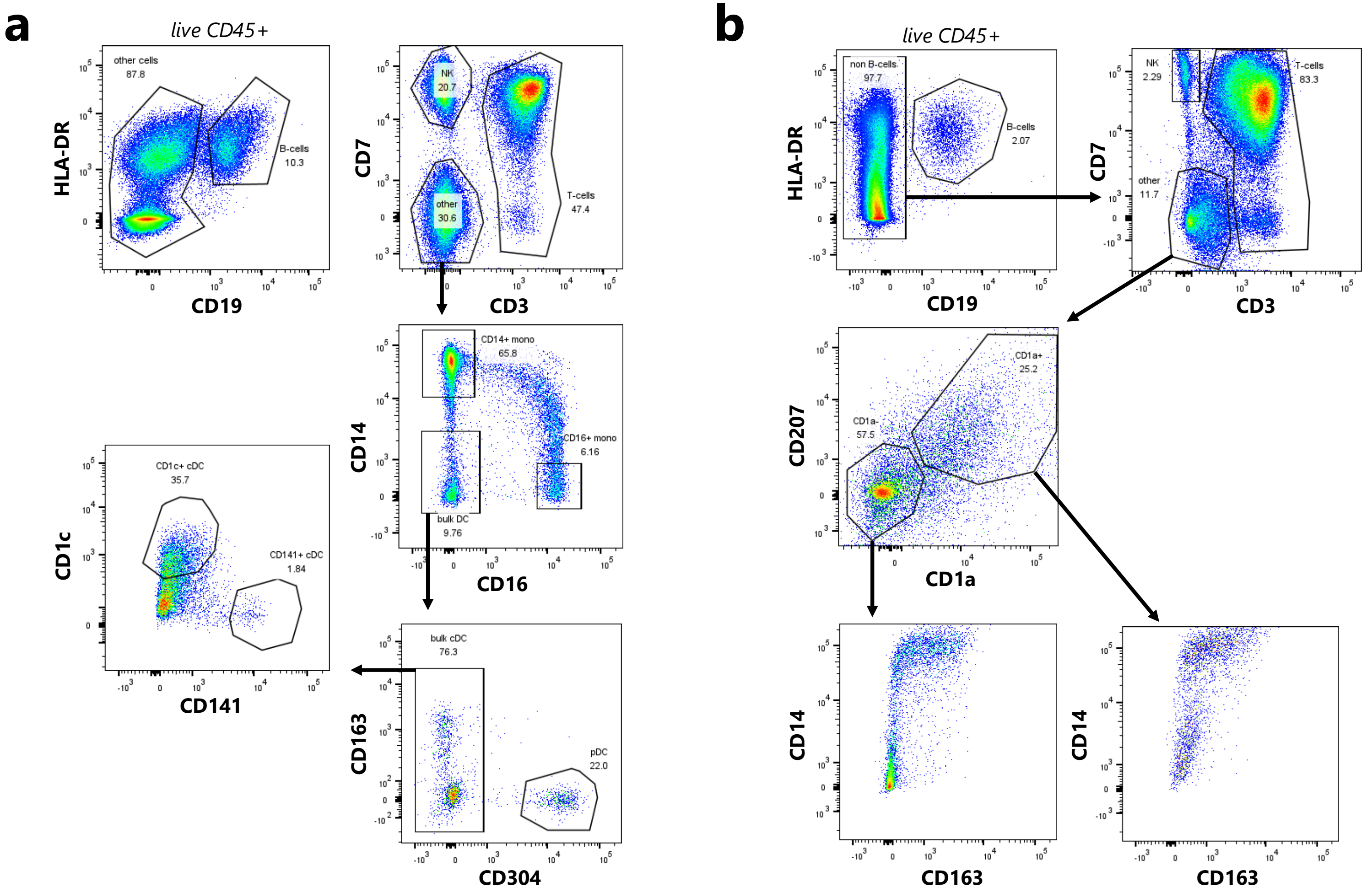

Gating strategies for hematopoietic cell type identification and sorting from peripheral blood **(a)** or lesional tissue **(b)**. Note that further separation of bulk classical dendritic cells (cDCs) into CD1c$^+$ or CD141$^+$ cDCs was performed only for Case #10. The following antibodies were used for flowcytometry-guided cell sorting (clone): CD45 (2D1), CD19 (SJ25C1), HLA-DR (G46-6), CD3 (SK7), CD7 (M-T701), CD14 (MOP9), CD16 (3G8), CD11c (B-Ly6), CD163 (GHI/61), CD304 (12C2), CD1a (HI149), CD207 (DCGM4) and CD141 (1A4). Except for CD163 and CD304 (both from Biolegend) and CD207 (from Beckman Coulter), all antibodies and 7AAD (live/death marker) were purchased from BD Biosciences.

Supplemental Figure 2. Reanalysis of sorted cell fractions to assess potential contamination

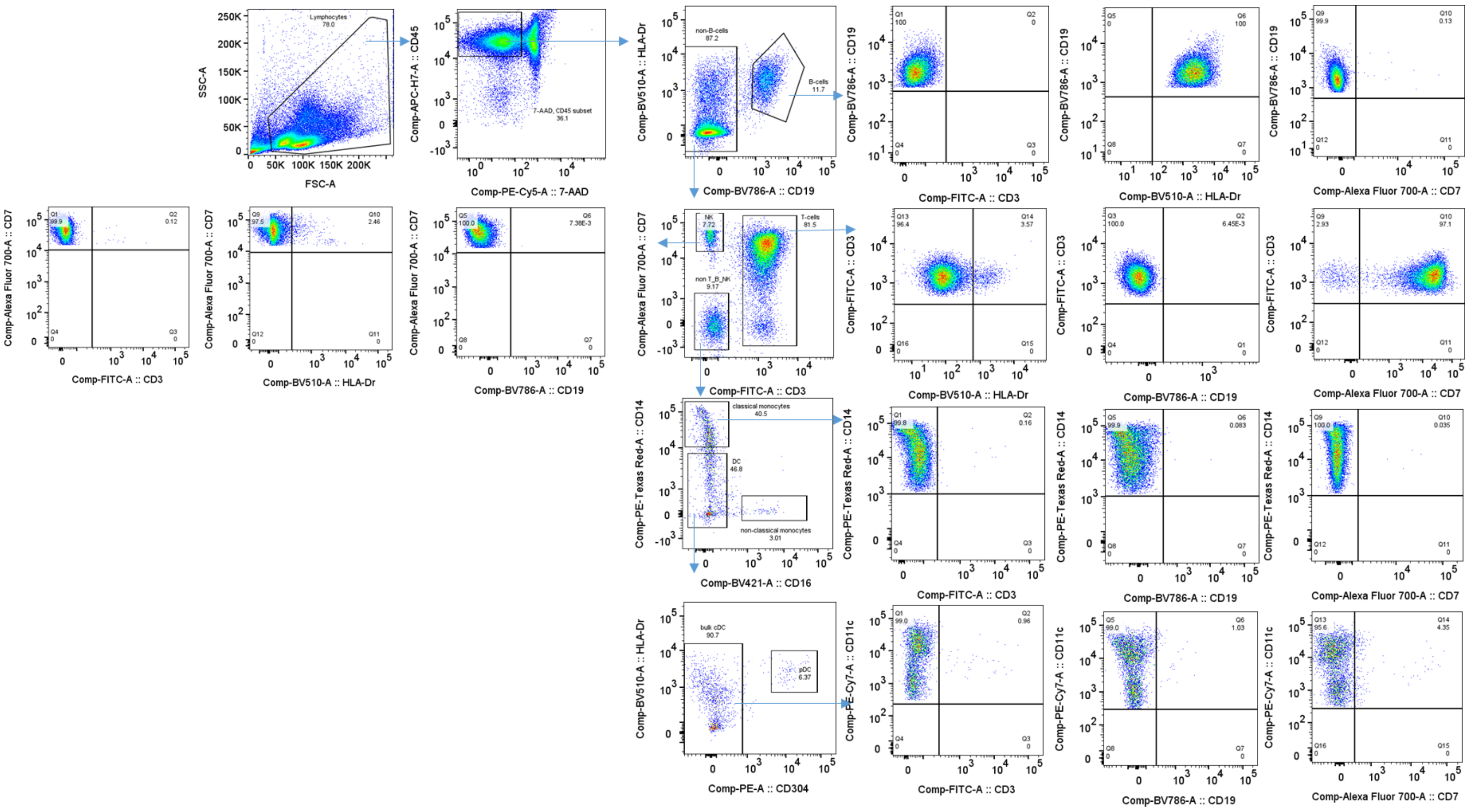

Reanalysis of CD3⁺ T cells, CD19⁺ B cells, CD3⁻ CD7⁺ NK cells, CD14⁺ CD16⁻ classical monocytes, and CD11c⁺ bulk dendritic cells isolated from a control PBMC sample, which was included in all sorting experiments conducted in this study. The vertical row of images in the center of the figure depicts cell gating, whereas the adjacent plots show the expression of sorted cell fractions for various lineage markers.

Supplemental Figure 3. Validation of droplet digital PCR assay specificity

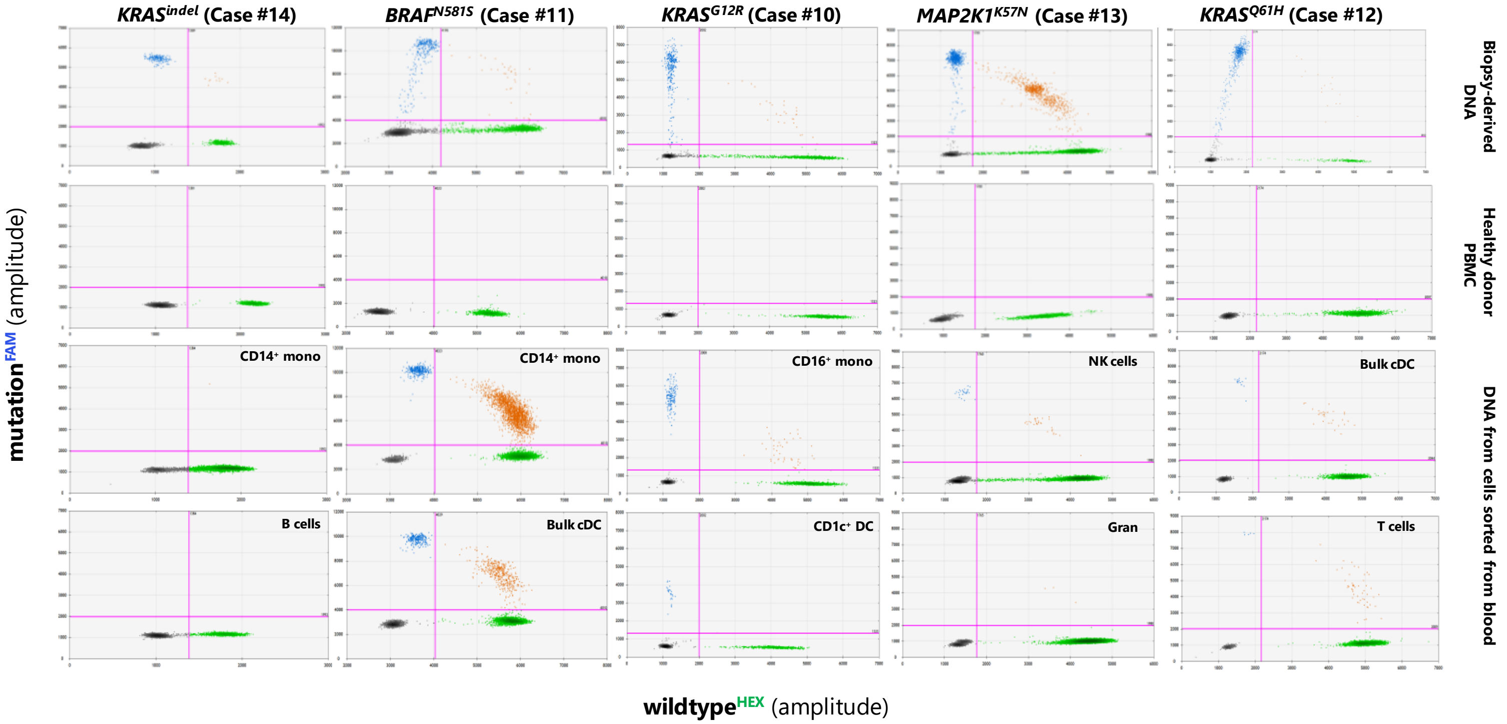

Exemplary results generated with five different ddPCR mutation detection assays. The upper horizontal row shows data generated on DNA extracted from the patient's histiocytosis biopsy or a *KRAS*^{Q61H} mutated rhabdomyosarcoma sample (Case #12), as positive control. The second horizontal row depicts results obtained with healthy donor PBMC-derived DNA (negative control). This negative control sample consists of a mixture of equal DNA amounts extracted from the PBMC of 100 different healthy blood bank donors. The third and fourth horizontal rows display results from two different flowsorted populations, as summarized in Figure 1a. The blue droplets contain mutation-specific amplicons, green droplets contain only wildtype sequence-specific amplicons, orange droplets contain both mutant- and wildtype-specific PCR products, and grey droplets contain no PCR product. The pink lines represent the thresholds, which were set on negative control results. Positivity for mutant alleles was defined as ≥3 droplets falling in the same range as blue and orange (mutated) droplets generated with the positive control sample. Theoretical sensitivity of mutation detection by ddPCR is 0.002% for samples reaching ≥400 ng DNA input, 0.005% for samples reaching 200 ng DNA input, and 0.01% for samples reaching 100 ng DNA input. Abbreviations: PB, peripheral blood; mono, monocytes; NK cells, Natural Killer cells; Gran, granulocytes. Footnotes: Bulk cDC means total CD11c^{+} DC fraction without CD11c^{-}CD304^{+} plasmacytoid DC. Gating is shown in supplemental Figure 1. The following commercially available assays (all from Bio-Rad) were used: *BRAF* p.V600E (dHsaMDV2010027), *MAP2K1* p.K57N (dHsaMDS38350127), *BRAF* p.N581S (dHsaMDS2511810), *KRAS* p.G12R (dHsaMDV2010009) and *KRAS* p.Q61H (dHsaMDV2010131). Primers and probe sequence of the ddPCR assay targeting the somatic *KRAS* insertion-deletion in Case #14 are available upon request from the corresponding author.

Supplemental Figure 4. Validation of droplet digital PCR assay reproducibility

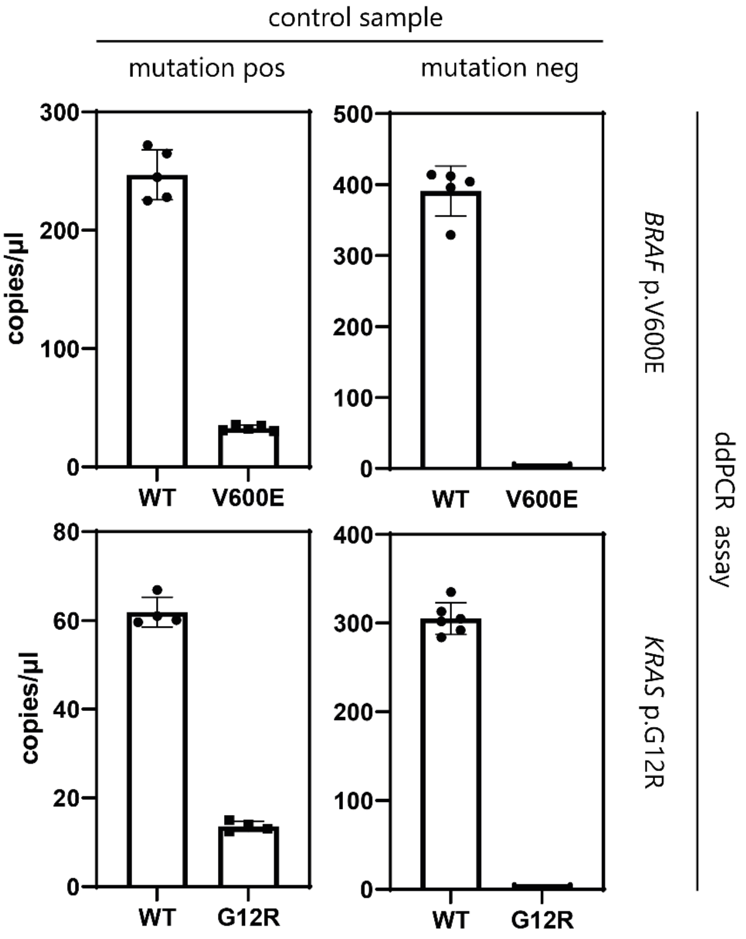

Repetitive testing of positive and negative control samples in *BRAF* p.V600E (top row) or *KRAS* p.G12R (bottom row) duplex ddPCR assays. Each dot represents a ddPCR result generated on a different time point with the relevant control sample.

Supplemental Figure 5. Consistent cellular distribution of *KRAS*^{G12R} over time

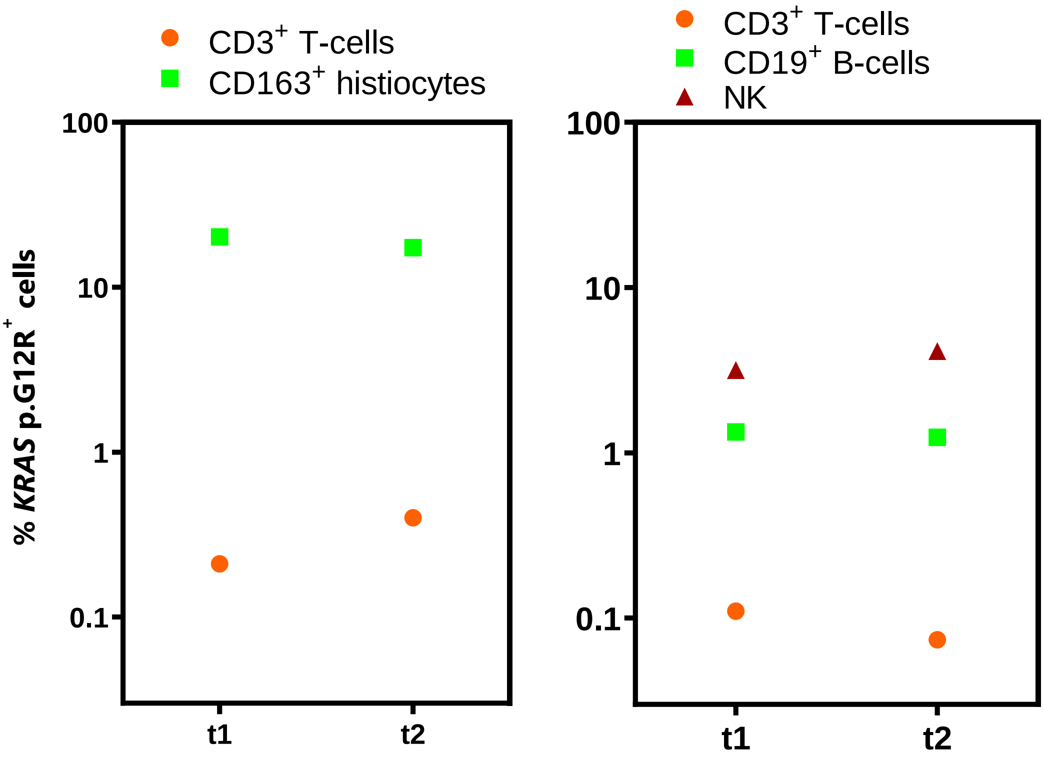

Assessment of *KRAS* p.G12R mutational burden in hematopoietic subsets isolated from resected xanthogranuloma (left) or peripheral blood (right) samples of Case #10, with a time interval between these samples of more than 1 year. Population gating for cell sorting was executed as depicted in Supplemental Figure 1.

Supplemental Figure 6. Detection of *$KRAS^{G12R}$* in erythroblasts isolated from blood in Case #10

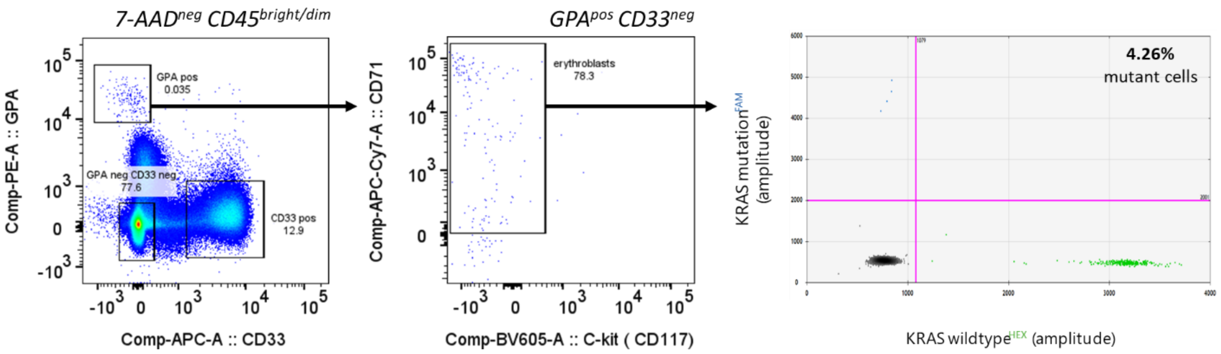

Abbreviations: GPA, glycophorin A; 7-AAD, 7-aminoactinomycin D

Supplemental Table 1. Details of included patients

Patient	Age$/Sex	Disease (extent)	Involved organs	Systemic therapy at sampling	Prior systemic therapy	Driver mutation
**L-group**						
1	45 y / F	LCH (SS)	Bone (unifocal: scapula)#	No	None	*BRAF* p.V600E
2	55 y / F	LCH (MS)	Bone, skin, lymph nodes, pituitary	No	None	*BRAF* p.V600E
3	66 y / F	ECD/LCH (SS)	Bone (multifocal: mastoid, spine, femurs)	No	None	*BRAF* p.V600E
4	59 y / M	ECD/LCH (MS)	Bone, dura, cardiovascular system, skin/soft tissue	No	None	*BRAF* p.V600E
5	25 y / M	ECD/LCH (MS)	Bone, skin/soft tissue	No	None	*BRAF* p.V600E
6	18 y / M	ECD (MS)	Bone, brain	No	Conventional*	*BRAF* p.V600E
7	49 y / F	ECD (MS)	Bone, brain, cardiovascular system, kidney	No	Vemurafenib	*BRAF* p.V600E
8	53 y / M	ECD (MS)	Bone, brain, cardiovascular system, kidney, skin/soft tissue	No	None	*BRAF* p.V600E
9	66 y / M	ECD (MS)	Bone, brain, cardiovascular system, kidney, skin/soft tissue	No	None	*BRAF* p.V600E
**C-group**						
10	34 y / M	AOX (SS)	Skin (multifocal)	No	None	*KRAS* p.G12R
11	46 y / M	AOX (SS)	Skin (multifocal)	No	None	*BRAF* p.N518S
12	49 y / M	AOX (SS)	Skin (multifocal)	No	None	*KRAS* p.Q61H
**R-group**						
13	52 y / F	RDD/ECD (MS)	Bone, GI tract, skin	No	Conventional** & cobimetinib	*MAP2K1* p.K57N
**M-group**						
14	4 m / M	LCS (SS)	Skin/subcutis (unifocal)	No	None	*KRAS* p.L19_Q22delinsFTIK

Abbreviations: LCH, Langerhans cell histiocytosis; ECD, Erdheim-Chester disease; AOX, adult onset xanthogranuloma; RDD, Rosai-Dorfman disease; LCS, Langerhans cell sarcoma; SS, single-system; MS, multisystem; GI, gastrointestinal; indel, insertion-deletion; NGS, next-generation sequencing. Footnotes: $ Age represents age at diagnosis; # This patient was diagnosed with unifocal bone LCH in the left proximal humerus. At time of blood sampling, the patient had a unifocal bone relapse in the left scapula. * Prednisolone, rituximab, cyclophosphamide, high-dose methotrexate, lenalidomide; ** (Methyl)prednisolone, dexamethasone, hydroxychloroquine, low-dose methotrexate, sirolimus, azathioprine.
